# General Medicine and Therapeutics

**Published:** 1893-10

**Authors:** 


### GENERAL MEDICINE AND THERAPEUTICS.

EDITED BY DR. LUTHER B. GRANDY.

### THE CARDIAC TONICS AND THEIR INDICATIONS.

At a meeting of the British Medical Association (*Revue Général de Clinique et de Thérapeutique*) Broadbent spoke of rest or moderate exercise, and of eliminative agents. He said that strophanthus seemed to be the typical heart-tonic, since it increases the power of the cardiac systole without modifying the contractility of the blood vessels. The author, who appears somewhat partial to this medicament, asserts that the failures reported at various times, regarding strophanthus in practical medicine, are due to the impurities of the preparations used. After strophanthus he places digitalis, adding that the former remedy sometime acts from the first or second dose. He says that digitalis increases the elimination of liquids, while caffeine enhances that of solids, and hence the utility of prescribing these two agents in combination in order to obtain the same good effect that a large dose of digitalis would produce. Lander Brunton answered that digitalis is at the same time a cardiac and a vascular tonic, but that it must not be forgotten that the principles of the drugs do not act in the same manner. *Digitaline*, he said, increases both the contractility of the cardiac muscular fiber and that of the coats of the blood vessels, while *digitoin* produces a contrary effect; thus, when in a cardiopathy there is an excessive constriction of the blood vessels, it is advisable to combine digitalis with *quasi*-dilator substances as nitrous ether, for example. Alongside of strophanthus Brunton places the oxy-spartein, which, like it, acts decidedly upon the heart, but not so much upon the vessels themselves. He believes that the best cardiac medicaments after digitalis are strophanthus and nitrous ether. The same author also insisted on the employment of muscular exercise in order to increase the arterial tension, but without putting strain upon the heart. In mitral disease, for instance, the rule is to have absolute rest, but in such cases massage is to be resorted to, a method by which the general nutrition may be increased without increasing cardiac muscular exertion. It is, then, useful to associate with massage passive exercise. Lastly, Brunton recommended, in the treatment of mitral disease for example, the administration of blue mass during the night and jalap by day.—*University Medical Magazine.*

---

### INTESTINAL ANTISEPSIS IN TYPHOID FEVER.

As the putrefactions and other symptoms and lesions referable to the intestines are a large factor in the morbid syndrome of the disease, the importance of intestinal antisepsis becomes obvious. Bouchard may almost be regarded as the founder of intestinal antisepsis. He rightly reasoned that remedies to reach the intestinal mucous membrane must be insoluble in the stomach. He early experimented with charcoal, magnesia, chalk, bismuth, naphthol, iodoform, naphthalin, salol, salicylate of bismuth from the point of view of intestinal antisepsis. He obtained seemingly good results from powdered charcoal. He gave a tablespoonful every two hours. His statistics based on more than three hundred cases showed a mortality reduced from twenty or twenty-five to fifteen per cent. William Jenner used charcoal before Bouchard in teaspoonful doses two or three times daily. He said that this had given him such satisfactory results that he resorted to no other remedies.

Bouchard, in his last published utterance, gives his preference to beta-naphthol, which he thinks is the ideal remedy, given in doses of seven and one-half grains in capsules every three hours, till the stools are completely deodorized and disinfected. Five grains each of bismuth salicylate and beta-naphthol may be given in capsules every three or four hours.

Salol, which may be given in five-grain doses every two hours, combined with an equal quantity of bismuth subnitrate in chalk mixture, is open to the objection of being soon spit up. However, it is believed to be able to correct the fetor of the stools, control abdominal pain, and arrest diarrhea. Oil of eucalyptus and oil of turpentine are both highly antiseptic. "More lives would be saved," says Prof. H. C. Wood, "if the oil of turpentine were more freely used in this disease." The use of camphor has been highly commended by Dr. Janeway of New York. Thymol, one grain and a half every six hours, has been used by Henry and Testi with good results.—*Boston Med. and Surg. Journal.*

---

### THE PROGNOSIS OF PHTHISIS.

Reisz analyzes two hundred and forty-seven cases of chronic pulmonary phthisis observed in his private practice. Of these, ninety recovered, seventy-six died, and eighty-one are still suffering from the disease. In his opinion the prognosis is especially bad when the phthisis is consecutive to an acute fever, as measles or pneumonia, or when it sets in with high fever; it is also a very bad symptom when the first stage of consumption is accompanied by fever and the glands of the neck become rapidly enlarged. In fatal cases the sputum either contains a multitude of tubercle bacilli or is quite devoid of them. These assertions are confirmed by the investigation of fatal cases as well as those which ended in recovery.—*Universal Medical Journal.*

---

### THE THERAPEUTIC ACTION OF SULPHONAL.

Kost (*Archiv. f. exp. Path. und Pharm.*, t. xxxi, fasc. I) reports nearly two hundred cases in which he employed sulphonal as a hypnotic. He prescribes it especially for combating nervous and febrile insomnia and that form accompanying certain physical disorders. The dose for a man is from two to three grammes, for a woman one gramme. Larger doses have given rise to serious after effects, such as vomiting, diarrhea, followed by obstinate constipation, heaviness of the head, paralysis, painful micturition and albuminuria. According to experiments instituted by Kost, three deleterious effects are to be attributed to alterations of the kidneys and not to educts of sulphonal, and they are the result only of large doses. He concludes with the following observations:

1. The maximum dose for a man is from two to three grammes, for a woman one gramme; these doses ought not to be exceeded except to combat intense excitement.

2. When one is obliged to continue the use of the drug for a prolonged period, intermissions of several days should be made from time to time.

3. The drug should be suspended as soon as such symptoms as nausea, vomiting or gastric pain appear.

4. Administered with these precautions, sulphonal is a harmless drug.—*Univ. Med. Mag.*

---

### ENTEROCLYSIS IN THE SUMMER DIARRHEA OF CHILDREN.

Müller (*Therapeutic Gazette*, Nos. 7 and 8, 1893) reports seventy-eight cases of summer diarrhea treated by enteroclysis in conjunction with other hygienic and remedial measures. His conclusions are as follows:

1. That intestinal irrigation may be considered a valuable adjunct in the methodical treatment of suitable cases of summer diarrhea.

2. That irrigation with cold or ice water will lower the temperature of the lower portion of the abdomen by direct refrigeration of the blood mass, and that the procedure is indicated when high temperature lasting for a considerable time endangers life by coagulating the cerebral fluid or cardiac protoplasm, or when accumulation of feces, mucus, etc., in the bowel causes a continued irritation of the mucous membrane.

3. *That the dangerous effects of the poisonous animal alkaloids are diminished or contracted or dissipated by irrigation.*

4. That the influence on the circulatory apparatus is shown by the change in the pulse, which becomes less frequent and stronger.

5. That systematic enteroclysis results in an amelioration of the symptoms and a shortening of the course of the affection, and will often overcome the semi-paralytic condition of different organs.

6. That the resistance which the fever offers to its reduction by this method is an index of the gravity or mildness of the case.

7. That alcoholic stimulants are of importance in the treatment of summer diarrhea in children.—*Univ. Med. Mag.*

### THE DIAGNOSIS OF TYPHOID FEVER.

Dr. V. Filipovich, of Odessa, has remarked that, in all the cases of typhoid fever that have come under his notice during the last two extensive epidemics in Odessa, there have been a peculiar induration and yellowish or orange tint in the prominent portions of the plantar and palmar surfaces, instead of the reddish color to be observed in healthy subjects, or the bluish tinge which those parts assume in a cyanotic patient. This yellowish appearance is to be accounted for by the enfeebled action of the heart, the incomplete filling of the capillaries, and the dryness of the skin in typhoid fever. The sign is so constant and so well marked, that Dr. Filipovich thinks that it should be looked upon as affording important assistance in the diagnosis of doubtful cases. That it is in no sense peculiar to the two epidemics that occurred in Odessa is shown by the observations of Dr. Skibnevski, who states that he invariably observed it during an epidemic in the neighborhood of Moscow.—*Lancet-Medical Record.*

---

### DIAGNOSIS AND TREATMENT OF GASTRIC ULCER.

J. Boas, of Berlin, Germany (*Medical Record*, N. Y., June 17, 1892), uses the usual means of arriving at a diagnosis and establishes it by the presence of localized pain in the epigastrium and upon the examination of the stomach contents. Pain, however, may be rarely absent and hyperacidity may be present in carcinoma when the growth is upon the base of an old ulcer. He has found after many observations that a dorsal point of tenderness exists almost as frequently as the epigastric point, and the point is so sharply circumscribed that for diagnostic purposes it has far more value than the point in the epigastrium. The point is found to the left of and on a level with the tenth to the twelfth dorsal vertebra, rarely higher or lower. It lies usually directly against the vertebra, rarely some distance from it. In a few instances the point is on both sides. In no other disease, and especially no other disease of the stomach, is the point found with equal constancy. In cases of colelithiasis there is a painful spot to the right of the twelfth dorsal vertebra. He uses an instrument called the "Ægesimeter," by which the pressure made can be measured. A painful point due to ulcer will not bear nearly as much pressure as one due to other causes. His treatment consist of rest in bed, limited diet and the administration of large doses of nitrate of silver in *solution.* He uses gr. iij to f℥iv of water at first and gradually increases the strength. The next bottle having gr. ivss and the third vi gr. to f℥iv. The dose is a tablespoonful diluted with a wine glass full of water and taken on an empty stomach. Some obstinate cases will not be relieved by this treatment. In these he gives no food by the mouth, but feeds by enemata. The yolk of two eggs, half pint of milk, one tablespoonful of wine or brandy and a tablespoonful of flour are warmed and given every three hours. The return to solid food is gradual, liquids being first given.—*Univ. Med. Mag.*

---

### Pneumonia.

Dr. H. C. Wood, in *Boston Medical and Surgical Journal,* says: There are pneumonias which are one thing, and there are pneumonias which, therapeutically, are another thing. Pneumonias therapeutically may have no more relation with one another than a pneumonia has with a dysentery, and are no more to be treated as the same disease, though we label them with the same name. When I speak to you about the treatment of pneumonias I do not mean the treatment of senile pneumonias nor of asthenic pneumonias; I mean the treatment of frank, sthenic, hard pneumonia, that comes upon a man as with the bound of a lion. I say here that I am certain that when our forefathers bled these cases they saved lives that now we lose. I am out of date perhaps; I am behind the times in one way, and I am ahead of the times in another way. The pendulum is beginning to swing a little towards venesection; but the thought I want to leave with you is that by means of this drug (veratrum viride) we can get all the good that comes from venesection. You bleed a man, you depress him, lower the immediate activity of the vital forces, but you also take away power, and by and by, when the fight comes, that man is exhausted. Venesection has the dangers that surround tartar emetic. Remember that the abdominal vessels in a man will after death contain all the blood in his body and not be full. We forget very often that it is the abdominal vessels that dominate the circulation. Some of you must have seen in the operation of ovariotomy a woman dying, blanched, upon the table, revived by a dash of hot water into the abdomen. The woman revives out of her syncope because hot water contracts the abdominal vessels. When we give a man veratrum viride we dilate his abdominal vessels—we bleed the man into his own belly. By the action of this drug we get the influence of venesection; but instead of withdrawing the blood entirely from the body, we put it in a reservoir, whence we can pour it back when the proper time comes.—*Med. Brief.*

---

### USE OF LIME WATER IN ARTIFICIAL INFANT-FEEDING.

One reason why cow's milk is not easily digested by infants is that the casein formed by the action of the curdling ferment of the gastric juice is dense and tough, while that formed from human milk is flaky. The addition of lime water to the cow's milk causes it to be precipitated in flakes also, and thus overcomes this disadvantage to a great extent. A tablespoonful of lime water to an ordinary bottle of milk is enough, and a little sugar of milk may be added to correct the taste of the lime water. Courant (*Revue de Thérapeutique Mèdico-Chir.*) has seen the best results follow this practice in gastric catarrh of children.—*The Therapeutic Gazette.*

---

FOR the night sweats of phthisis pulmonalis, the following was one of Fothergill's favorite prescriptions:

Atropia sulph., gr. 1—50.
Morphine sulph., gr. 1—4.
Aqua camphoris, oz. i.
M. Sig.—Teaspoonful at bedtime.

